# Performance of two different artificial intelligence (AI) methods for assessing carpal bone age compared to the standard Greulich and Pyle method

**DOI:** 10.1007/s11547-024-01871-2

**Published:** 2024-08-20

**Authors:** Davide Alaimo, Maria Chiara Terranova, Ettore Palizzolo, Manfredi De Angelis, Vittorio Avella, Giuseppe Paviglianiti, Giuseppe Lo Re, Domenica Matranga, Sergio Salerno

**Affiliations:** 1 Dipartimento di Diagnostica per Immagini Policlinico, Università degli Studi di Palermo, Via del Vespro 127, 90127 Palermo, Italy; 2 UOC Radiologia Pediatrica Dipartimento di Diagnostica per Immagini e Interventistica, ARNAS, Ospedali Civico, Di Cristina Benfratelli, Palermo, Italy; 3 Dipartimento Promozione della Salute, Materno-Infantile (PROMISE), Università Di Palermo, Palermo, Italy

**Keywords:** Artificial intelligence, Bone age, Carpal age, Age assessment

## Abstract

**Purpose:**

Evaluate the agreement between bone age assessments conducted by two distinct machine learning system and standard Greulich and Pyle method.

**Materials and methods:**

Carpal radiographs of 225 patients (mean age 8 years and 10 months, SD = 3 years and 1 month) were retrospectively analysed at two separate institutions (October 2018 and May 2022) by both expert radiologists and radiologists in training as well as by two distinct AI software programmes, 16-bit AI^tm^ and BoneXpert® in a blinded manner.

**Results:**

The bone age range estimated by the 16-bit AI^tm^ system in our sample varied between 1 year and 1 month and 15 years and 8 months (mean bone age 9 years and 5 months SD = 3 years and 3 months). BoneXpert® estimated bone age ranged between 8 months and 15 years and 7 months (mean bone age 8 years and 11 months SD = 3 years and 3 months). The average bone age estimated by the Greulich and Pyle method was between 11 months and 14 years, 9 months (mean bone age 8 years and 4 months SD = 3 years and 3 months). Radiologists’ assessments using the Greulich and Pyle method were significantly correlated (Pearson’s *r* > 0.80, *p* < 0.001). There was no statistical difference between BoneXpert® and 16-bit AI^tm^ (mean difference = − 0.19, 95%CI = (− 0.45; 0.08)), and the agreement between two measurements varies between − 3.45 (95%CI = (− 3.95; − 3.03) and 3.07 (95%CI − 3.03; 3.57).

**Conclusions:**

Both AI methods and GP provide correlated results, although the measurements made by AI were closer to each other compared to the GP method.

## Introduction

Bone growth encompasses alterations in bone size, shape, and mineral density. This occurs through the activity of primary and secondary centres of ossification, the bone formed from the first centre is known as the diaphysis, and from the second, the epiphysis, respectively. In these centres, cartilage gradually transforms into bone tissue. This progression continues as long as cartilage remains present in the growth plate, also known as the epiphyseal plate. Upon completion of bone development, the epiphyseal plate undergoes ossification, indicating fusion between the diaphysis and epiphysis [1]. Bone age serves as a marker of bone maturity making its assessment common in paediatric radiology. It aids in evaluating growth, maturity, and diagnosing and managing various paediatric disorders, including endocrinological, orthodontic, and orthopaedical conditions. Accurate assessment relies on understanding the shape and maturity level of primary and secondary ossification centres and their fusion times [2, 3]. The two primary applications of skeletal age assessment are the identification of growth disorders and the estimation of eventual adult height. From a legal standpoint, bone age assessment could play a role in determining whether an individual is a minor when official documents are unavailable. However, according to the European Society of Paediatric Radiology (ESPR), evaluating the bone age of the hand and wrist alone to determine chronological age is not recommended because it is not possible to overcome the large biological variation or the statistical problems associated with endpoint maturation of the wrist [4].

Over the past decades, various methods have been utilized including the Greulich–Pyle (GP), the Gilsanz–Ratibin, and the Tanner–Whitehouse (TW) methods. The GP and the Gilsanz–Ratibin methods are atlas-based, comparing the patient's radiograph to standard atlas radiographs and assigning the nearest bone age [1, 5, 6]. Conversely, TW employs a scoring method, staging specific radiographic regions of interest (ROI) of the radius, ulna, and short bones, to derive a final score converted into bone age [7]. Greulich and Pyle's Radiographic Atlas of Skeletal Development of the Hand and Wrist (G&P) presents left-hand radiographs chosen as sex-specific developmental benchmarks across various ages. The atlas includes tables of mean skeletal ages and standard deviations (SD), categorized by chronological ages and sex, facilitating assessments of skeletal maturity in children [6]. Greulich and Pyle curated representative radiographs to correspond with each age group in the atlas. By comparing these standards with radiographs from hundreds of typically developing children of similar ages, they calculated standard deviations for each age group. Despite GP's creation using radiographs from the forties and fifties, it continues to be widely used in clinical practice, albeit requiring manual processing, and is applicable to multi-ethnic populations in developed countries [8, 9]. The manual approach of the G&P method involves reviewing images and text, consulting data tables, and performing basic calculations to assess skeletal age against chronological age. This manual process can slow down diagnostic workflows and increase the risk of both observer and mathematical errors [8].

BoneXpert® (Visiana, Holte, Denmark) is a fully automated system that operates without the need for manual verification by an expert, introduced in 2009. This software adopts a more nuanced approach, examining radiographs of the left hand and wrist to evaluate bone age (BA). It evaluates 13 bones, including the ulna, radius, and 11 short bones in fingers 1, 3, and 5. Bone morphology, density scores, and textural features serve as critical parameters for this algorithm to discern and distinguish bone structures. The radiograph analysis is segmented into three successive layers. Initially, the software identifies bones of interest by applying active appearance models. Subsequently, it determines and verifies the bone age for each identified bone. In the final stage, the software converts the computed BA values into GP or TW BA values [10]. Recently, another bone age application called Physis® (developed by 16-bit AI™, Toronto, Canada) has been introduced [11]. It analyses left-hand and wrist radiographs, providing the predicted bone age. This application, which was the winner of the 2017 RSNA Paediatric Bone Age Challenge, attained a concordance correlation coefficient (CCC) of 0.991 when compared to the ground truth determined by radiologists along with a mean absolute difference of 4.265 months [12].

In our study, we aimed to compare agreement between measurements obtained using the standard GP method, 16-bit AI^tm^ software (free version begin 2022), and BoneXpert® system.

## Materials and methods

### Patients’ selection

The sample included 225 retrospective consecutive patients between 11 months and 16 years and 1 months, with a mean age 8 years and 10 months (SD = 3 years and 1 month), comprising 120 males and 105 females. They underwent clinically indicated radiographs of the left hand and wrist between October 2018 and May 2022 at two institutions, conducted under blinded conditions.

### Bone age assessment

Both the experts, with over 20 years of experience, and three radiology residents, with varying levels of experience, analysed each radiograph. A different level of experience was chosen to assess intra-reader variability. They were aware of the patient's sex but not their age, estimating bone age based on GP tables. Subsequently, we utilized demo versions of the last available release of BoneXpert® 3.2.2. The software automatically extracts the BA of 13 bones (radius, ulna, and 11 short bones in fingers 1, 3, and 5) directly from Digital Imaging and Communications in Medicine (DICOM) images sent on AI software server. Initially, it reconstructs and validates bone margins, calculates the BA of each bone and the total intrinsic BA, and then converts this value into GP or TW values. The software also automatically rejects suboptimal images. Subsequently, the software sends back a new labelled DICOM file to the Picture Archiving and Communication System (PACS) [13]. Next, we exported the original DICOM images from the PACS to JPEG format with the maximum allowed quality and submitted them to the free version of 16-bit AI^tm^ software (16-bit, Toronto, Canada).

The software, as described on the website, employs a deep convolutional neural network (DCNN) trained on 12.612 paediatric radiographs, as stated by company in early 2022. It requires the radiograph and the patient's sex to assess the estimated bone age and the chronological age to assess the standard deviation. Upon uploading the JPEG image to the server, it automatically resizes it to 500 × 500 pixels before analysis. After a few seconds, the software provides the bone age, the standard deviation, and the inference time [11, 12].

## Statistical analysis

We conducted a repeated measures study involving four readers (one expert and three radiology residents) to evaluate the determined sample of radiographs. After assessing normality using the Shapiro–Wilks test, we calculated the Pearson’s correlation and the one-way ANOVA to assess reproducibility among the four readers. Subsequently, we determined the GP bone age as the mean of the four repetitions. Following another assessment of normality using the Shapiro–Wilks test, the chronological age and the bone age as measured through 16-bit AI^tm^, BoneXpert®, and GP were summarized as mean and standard deviation. To compare the measurements obtained from 16-bit AI^tm^, BoneXpert®, and GP, we constructed three Bland–Altman plots. Each pair of measurements was represented as coordinates on a Cartesian system with their difference on the y-axis and their mean on the x-axis. A negative difference on the y-axis (positive difference) indicated bias between the two measurements (16-bit AI^tm^, BoneXpert®, and GP), with the first measurement systematically below (above) the second measurement. The bias was considered statistically significant if the line of equality (zero difference) fell outside the 95% confidence interval (CI) of the mean difference. Points outside the 95% limits of agreement indicated a pair of measurements with significant disagreement.

## Results

The Shapiro–Wilks test suggested Gaussian distribution for all 16-bit AItm bone age (*p* = 0.1789), GP bone age (*p* = 0.1730), BoneXpert® bone age (*p* = 0.0956), and chronological age (*p* = 0.5041) 0.16-bit AI^tm^ bone age ranged from 1 year and 1 month to 15 years and 8 months, with a mean bone age 9 years and 5 months (SD = 3 years and 3 months). BoneXpert® bone age ranged between 8 months and 15 years and 7 months, with a mean bone age 8 years and 11 months (SD = 3 years and 3 months). The average GP bone age was between 11 months and 14 years and 9 months, with a mean bone age 8 years and 4 months (SD = 3 years and 3 months) (Table 1). The less variability there is among these values—the mean, age range, and standard deviation (SD)—the greater the agreement between the evaluation systems. Furthermore, more defined and narrower age ranges and SDs imply greater precision in bone age assessment. The GP assessments by the four radiologists were significantly correlated (Pearson’s *r* > 0.80, *p* < 0.001). The heterogeneity among the readers was not statistically significant (ANOVA *p* = 0.952).

**Table 1:** Descriptive statistics of age and bone age estimated through GP, BoneXpert®, and 16-bit AI in a sample of 225 children

	Mean	SD	Min	Max
Chronological age	8 years and 10 months	3 years and 1 month	11 months	16 years and 1 month
GP^a^	8 years and 4 months	3 years and 3 months	11 months	14 years and 9 months
16-bit AI	9 years and 5 months	3 years and 3 months	13 months	15 years and 8 months
BoneXpert®	8 years and 11 months	3 years and 3 months	8 months	15 years and 7 months

^a^Average of all readers

The SD of the difference between bone age and chronological age was 15 months for BoneXpert®, 16 months for GP, and 22 months for 16-bit AI^tm^.

**Fig. 1:**
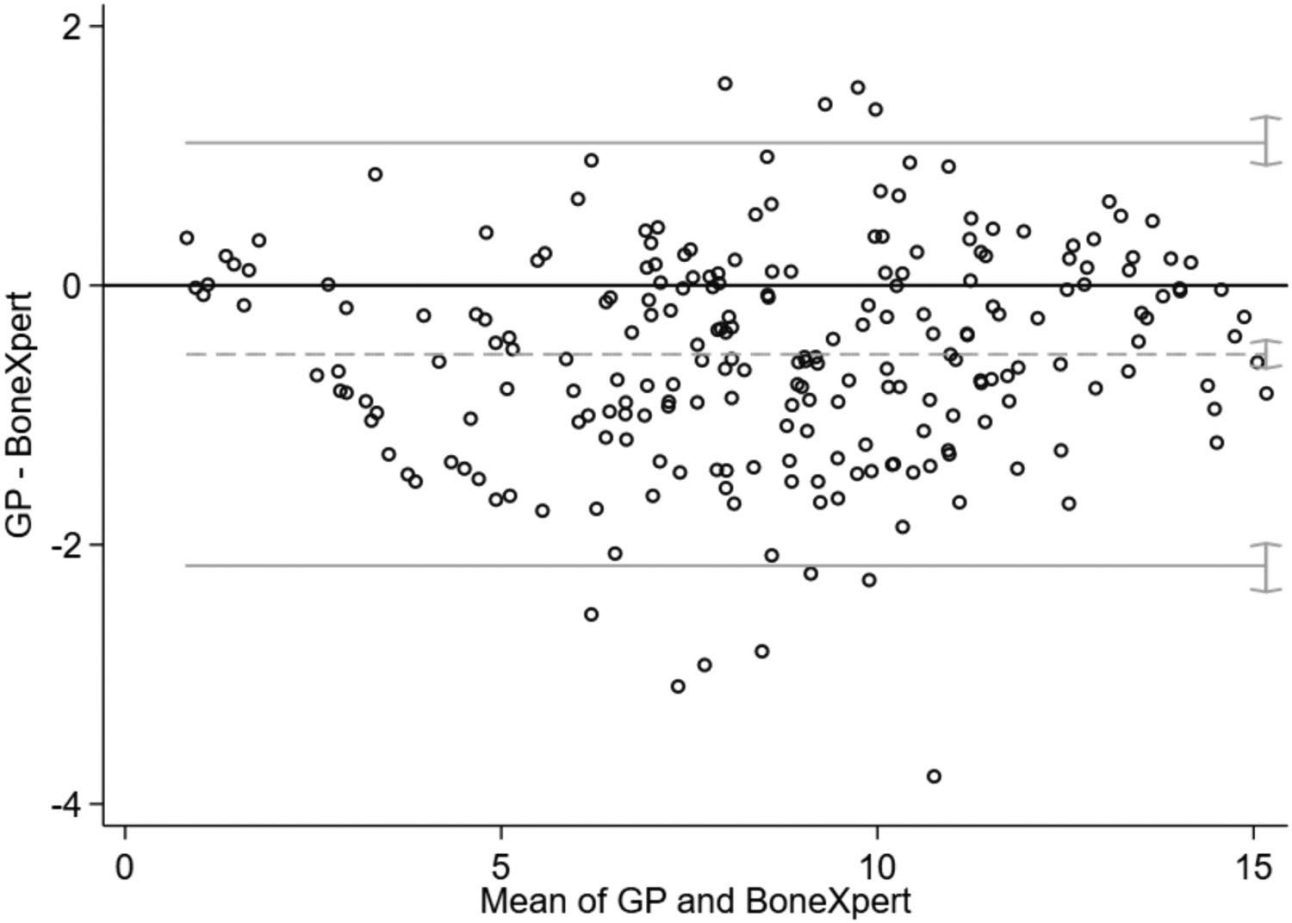
The Bland–Altman graph to assess the accuracy of GP vs BoneXpert®.^1^ *Note* ^1^On the x-axis: the mean of GP and BoneXpert®, on the y-axis: the difference GP–BoneXpert®. The y_zero_ line indicates unbiasedness. Grey lines indicate the bias (dashed) and 95% limits of agreement between two methods (continuous). Grey segments on the right of grey lines indicate 95% confidence intervals for the bias and for the limits of agreement. GP is the average assessment of the four readers

**Fig. 2:**
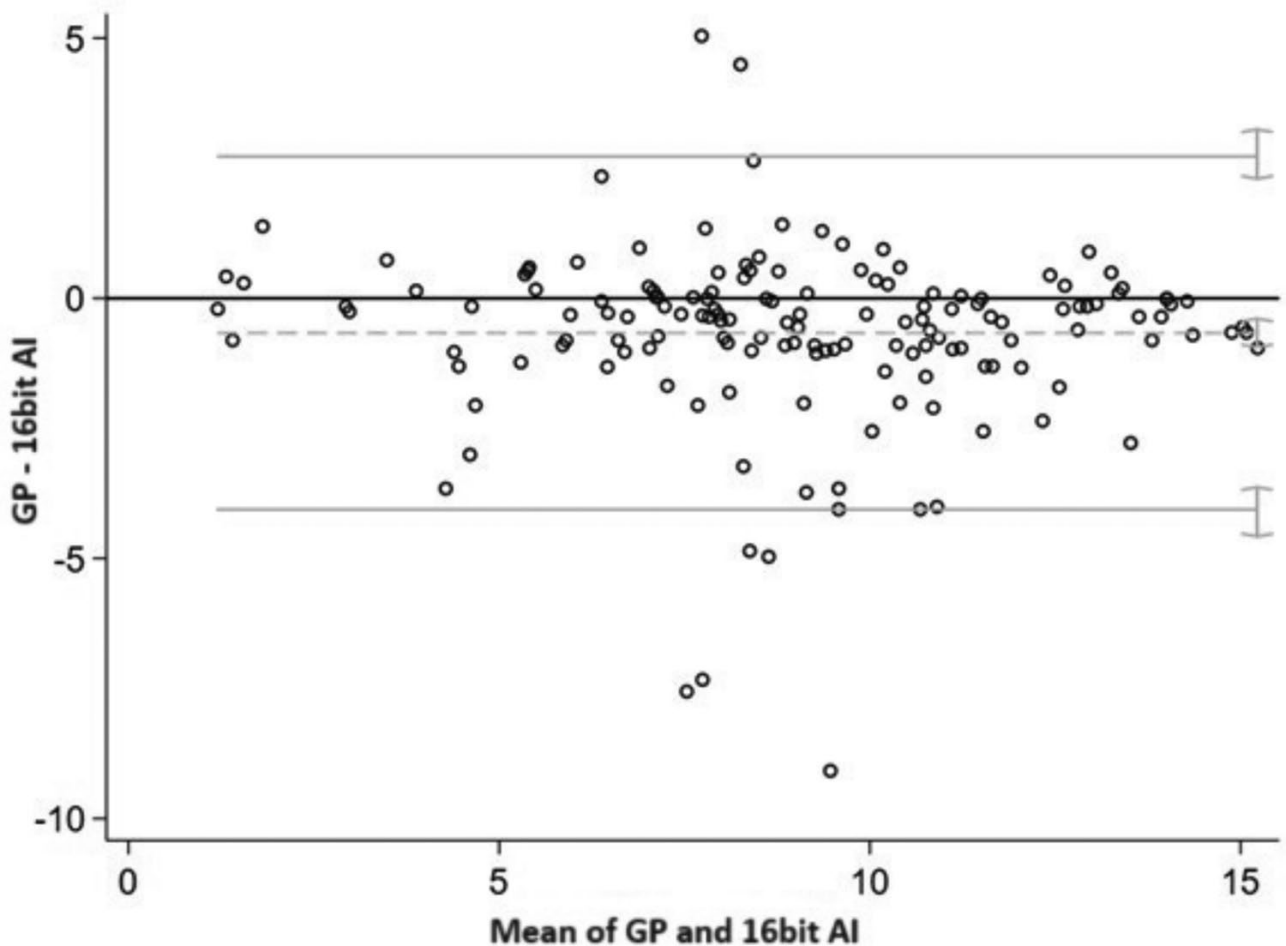
The Bland–Altman graph to assess the accuracy of GP vs 16-bit AI.^1^ *Note* ^1^On the x-axis: the mean of GP and 16-bit AI, on the y-axis: the difference GP–16-bit AI. The y_zero_ line indicates unbiasedness. Grey lines indicate the bias (dashed) and 95% limits of agreement between two methods (continuous). Grey segments on the right of grey lines indicate 95% confidence intervals for the bias and for the limits of agreement. GP is the average assessment of the four readers

**Fig. 3:**
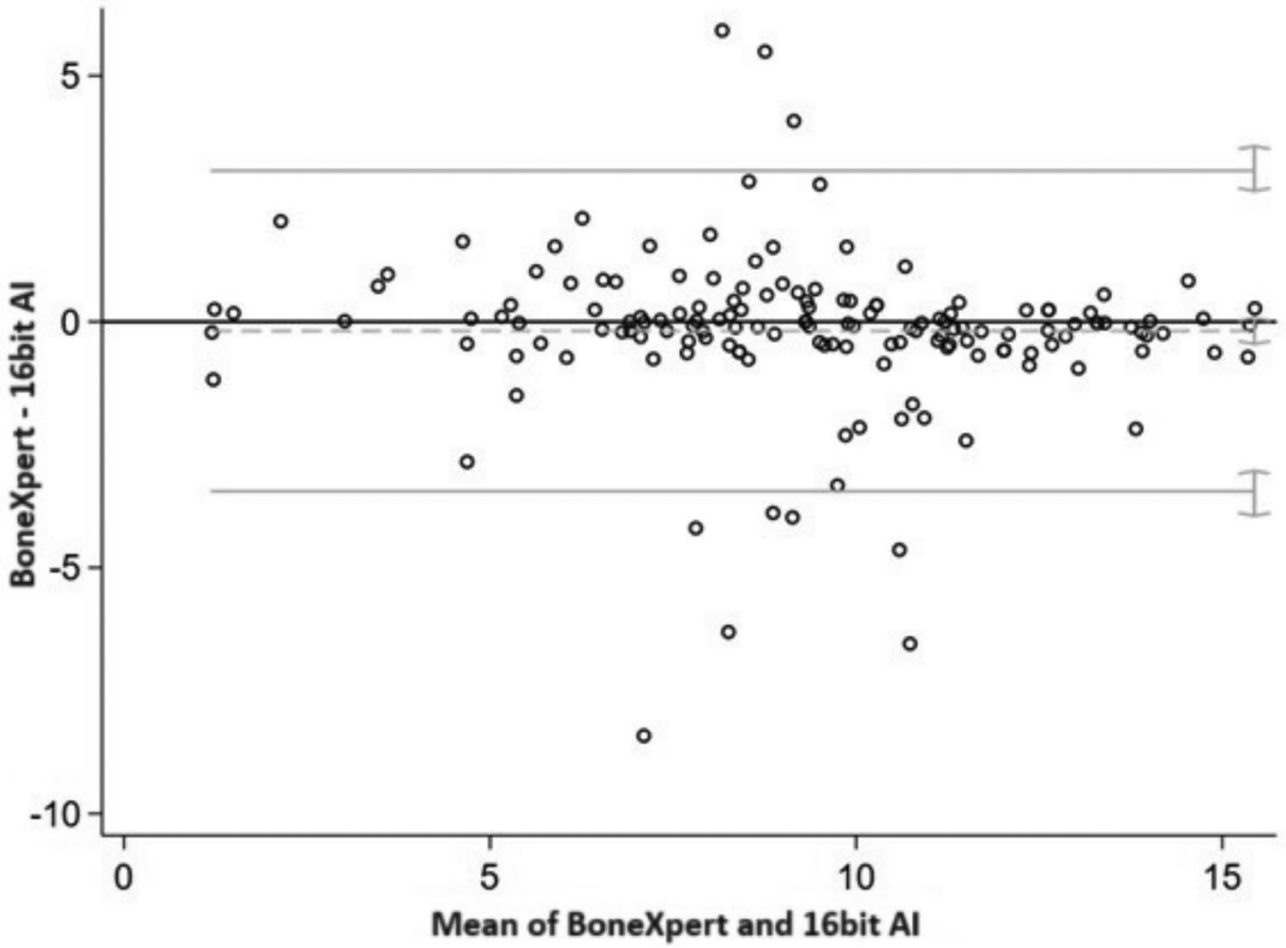
The Bland–Altman graph to assess the accuracy of BoneXpert® vs 16-bit AI.^1^ *Note* ^1^On the x-axis: the mean of BoneXpert® and 16-bit AI, on the y-axis: the difference BoneXpert®–16-bit AI. The y_zero_ line indicates unbiasedness. Grey lines indicate the bias (dashed) and 95% limits of agreement between two methods (continuous). Grey segments on the right of grey lines indicate 95% confidence intervals for the bias and for the limits of agreement. GP is the average assessment of the four readers

GP underestimated the bone age compared to BoneXpert® (mean difference = − 0.95, 95%CI = (− 0.64; − 0.42)) with a mean difference of 7 months, and the agreement between two measurements varied between − 2.16 (95%CI = (− 2.36; − 1.99) and 1.10 (95%CI 0.93; 1.30) (Fig. 1). Similarly, GP underestimated the bone age compared to 16-bit AI^tm^ (mean difference = − 0.67, 95%CI = (− 0.94; − 0.39)) with a mean difference of 1 year and 1 month, and the agreement between two measurements varied between − 4.06 (95%CI = (− 4.58; − 3.63) and 2.73 (95%CI = 2.30; 3.25) (Fig. 2). There was not systematic difference between BoneXpert® and 16-bit AI^tm^ with a mean difference of 7 months (mean difference = − 0.19, 95%CI = (− 0.45; 0.08)), and the agreement between two measurements varied between − 3.45 (95%CI = (− 3.95; − 3.03) and 3.07 (95%CI − 3.03; 3.57) (Fig. 3).

## Discussion

First, it is essential to acknowledge the intrinsic limitation of bone age assessment based on hand and wrist radiographs which is the wide range of standard deviations typically observed, ranging between 4 and 6 years or possibly higher. Factors such as nutrition, medications, and ethnic variations can influence the skeletal maturation. This limitation also explains why relying solely on this type of assessment alone is unreliable for determining whether a person is younger or older than 18 years old, a common issue we often encounter. In fact, the European Society of Paediatric Radiology (ESPR) recently stated that using of bone age of the hand alone cannot be recommended as a tool for chronological age determination [4].

Despite this limitation, bone age estimation is still widely used for medical reasons for various clinical purposes, mainly for assessing growth disorders. In our study, we analysed the accuracy and efficiency of two different AI software for bone age assessment and compared them with the GP method and among themselves. We used chronological age as the reference standard.

Our analysis reveals that both GP and AI methods showed a mean difference, with the highest variability observed in measurements made with the GP method, possibly due to operator-dependent evaluations, irrespective of experience, compared to objective computer-based estimations. Additionally, time needed for non-AI reading is higher as demonstrated by Kim et al. [14] and AI systems, such as BoneXpert, substantially decrease the time required for reporting bone age determinations [15].

Intra-readers variability is significantly reduced using both AI systems [16], and our results are consistent with the study by Gerges M et al., which demonstrated that the automated algorithm (specifically 16-bit AItm software) produced values in line with the GP method while also reducing analysis time [16]. Another study has demonstrated that BoneXpert® provides a highly accurate automated assessment of BA and may improve efficiency in clinical practice by reducing reading times without compromising accuracy compared to the Greulich–Pyle method [13]. In our experience, intra-reader variability affects both young and experienced radiologists, and this variability decreases using both AI systems. The use of AI systems allows for reliable results that are comparable to the manual GP method, with a reduction in intra-operator variability and in a faster manner.

Our study has some limitations: Firstly, we lacked access to the clinical history of our patients, meaning that the chronological age may differ from the bone age due to various clinical conditions. However, this holds true for all three bone age assessment systems used. Moreover, a recent study from Hi P.H. et al. highlighted that the 16-bit AI^tm^ software does not recognize wrong inputs, such as photos of flowers or chest radiographs, emphasizing the importance of the operator in managing AI [17]. Thirdly, we used a free demo version of 16-bit AI^tm^ software, and the commercially available licenced version now registered by Health Canada® could potentially be more developed and show an even greater correlation with the GP method and BoneXpert®.

## Conclusions

Both AI methods and GP provide correlated results, although the measurements made by AI were closer to each other compared to the GP method. The use of AI systems allows for a quicker assessment of bone age, offering results comparable to the GP method, regardless of the operator's experience.
